# Mutational analysis of fructose-1,6-bis-phosphatase FBP1 indicates partially independent functions in gluconeogenesis and sensitivity to genotoxic stress

**DOI:** 10.15698/mic2017.02.557

**Published:** 2017-02-01

**Authors:** Ali Ghanem, Ana Kitanovic, Jinda Holzwarth, Stefan Wölfl

**Affiliations:** 1 Institute of Pharmacy and Molecular Biotechnology, Heidelberg University, Heidelberg, Germany.

**Keywords:** FBP1, S. cerevisiae, MMS, genotoxicity, DNA-damage, gluconeogenesis

## Abstract

Fructose-1,6-bisphosphatase (*FBP1)* is a key enzyme in the evolutionary conserved pathway of gluconeogenesis. We had shown in an earlier study that *FBP1* is involved in the response and sensitivity to methyl-methanesulfonate (MMS)-induced DNA damage in yeast. In the work presented here we performed an alanine screen mutational analysis of several evolutionary conserved amino acid residues of *FBP1*, which were selected based on conserved residues and structural studies of mammalian and yeast homologues of *FBP1*. Mutants were examined for enzymatic activity, and yeast cells expressing these mutants were tested for growth on non-fermentable and MMS-containing media. The results obtained support predicted vital roles of several residues for enzymatic activity and led to the identification of residues indispensable for the MMS-sensitizing effect. Despite an overlap between these two properties, careful analysis revealed two mutations, Asn75 and His324, which decouple the enzymatic activity and the MMS-sensitizing effect, indicating two distinctive biological activities linked in this key gluconeogenesis enzyme.

## INTRODUCTION

Fructose 1,6 bisphosphatase (*FBP1*) has been known as the key-enzyme for gluconeogenesis, it irreversibly mediates the splitting of fructose 1,6 bis-phosphate into fructose 6 phosphate and inorganic phosphate. While gluconeogenesis shares most of its enzymes with glycolysis, Fbp1p is the only distinctive gluconeogenesis enzyme in the upper part of the gluconeogenesis pathway, through which the flux of this pathway can be regulated.

Gluconeogenesis has been thoroughly investigated under normal physiological conditions and in the adaptation to different nutrient conditions, and its importance in securing vital glucose homeostasis has been understood. However, the role of gluconeogenesis regulation and its effects in other conditions as for example during the development of tumours have long been overlooked 1.

The preference towards aerobic glycolysis as a major energy supply source is a common metabolic hallmark of both cancerous cells and many unicellular organisms including the yeast *S. cerevisiae*, both being aerobic fermenters with the major fermentation product being lactate in cancerous cells and ethanol in *S. cerevisiae *2,3,4.

The induced glycolysis in cancers known as ‘The Warburg Effect’, its underpinnings and advantages for tumours form the cornerstone of our comprehension of cancer metabolism; nevertheless several additional aspects of tumour metabolism have remained in the shadow for decades 5. In the last few years, a decisive role of FBP1-repression in the initiation of several tumour types has been described; metabolically, the loss of FBP1 increased glycolytic flux, and decreased respiration 6,7,8; morphologically, FBP1-loss has been demonstrated to be essential for the epithelial to mesenchymal transition (EMT), an essential morphological reprogramming step of epithelial tumor cells required for the dissemination of tumor cells from primary tumors, subsequently leading to the formation of metastasis 7.

Aerobic fermentation is also a hallmark of the yeast *S. cerevisiae* when grown in glucose rich medium and tightly controlled by nutrient sensing mechanisms 9,10. Others and we have shown that there is a close link between metabolism and sensitivity to DNA damage in yeast cells 11,12.

We had shown that expression of *FBP1* is induced in response to treatment with the genotoxic agent methyl-methanesulfonate (MMS) and that sensitivity of the yeast *S. cerevisiae* to MMS treatment is correlated with *FBP1* expression 13. High levels of Fbp1p obtained by overexpression increased sensitivity, while lack of *FBP1* reduced sensitivity to MMS, which suggests a regulatory role for this enzyme in DNA damage response and in the resulting oxidative stress conditions 13. This multi-faceted impact in addition to the high evolutionary conservation of several regulatory mechanisms and their related functions between the yeast and mammalian homologues, make yeast an attractive model-system to further probe and understand the multiple effects of *FBP1*. Here we present and discuss the results from a mutational screening of several evolutionary conserved amino acid residues of Fbp1p, predicted to be important for catalysis and/or allosteric regulation of this enzyme. We assessed the impact of these mutations on enzymatic activity, and further investigated their effect for growth on non-fermentable medium and sensitivity of yeast cells harbouring these mutant enzymes towards MMS treatment. In our study, we identified several indispensable residues for catalysis and MMS sensitization. The results obtained range from complete inactivation of the enzymatic activity of Fbp1p to partially deactivating mutations. Moreover, two mutations have been found, Asn75 and His325, which decouple catalysis from MMS-sensitization.

## RESULTS

### Selection of amino acid residues for mutational analysis

**Figure 1:**
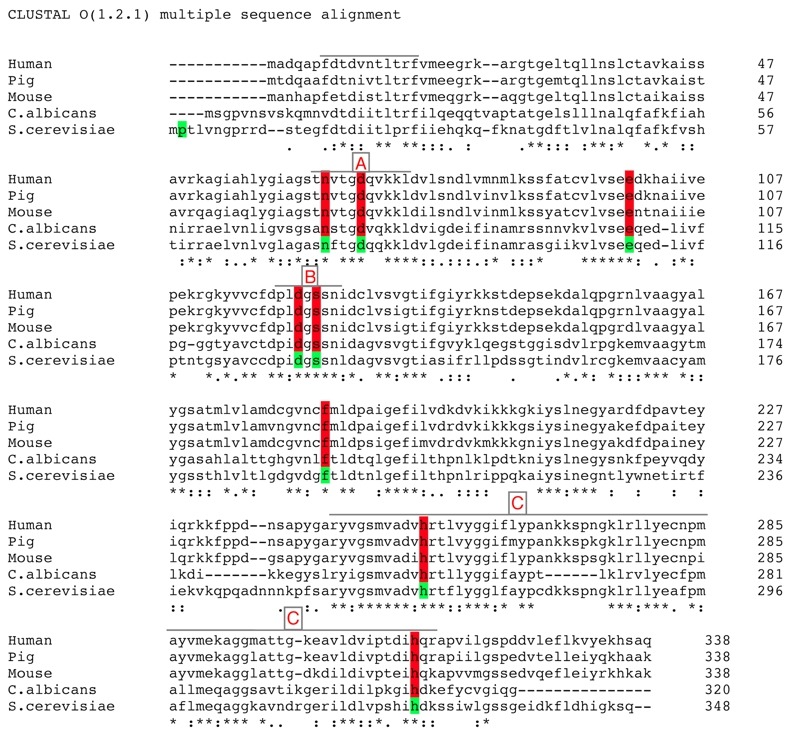
FIGURE 1: Multiple global-alignment of *FBP1* amino-acid sequences of *Saccharomyces cerevisiae* and several species using the online-available ClustalW2 multiple sequence alignment tool, provided by EMBL-EBI. Identical residues are marked with (|) ; similarities (:) and totally different ones with (.). Residues included in the mutational analysis are highlighted. Three regions (a, b and c) of sequence homology are indicated: **(A)** representing the mostly conserved part of the regulatory loop, **(B)** the conserved metal binding site, and **(C)** the largest homology domain on the c-terminus containing the active site.

We prepared a small library of mutated yeast *fbp1* expression cassettes, each with a single point mutation, changing one evolutionary-conserved residue. Global alignment of the amino acid sequence of Fbp1p of *Saccharomyces cerevisiae* against that of human *Homo sapiens*, pig *Sus scrofa*, mouse *Mus musculus* and another yeast *Candida albicans* shows around 46% identity (Fig. 1) 14,15. This allows a large choice of conserved residues to be manipulated. Therefore, we employed data from structural and functional studies performed on Fbp1p from yeast and mammalian homologues to identify residues that are more likely to have a key role in catalysis and/or allosteric regulation.

A general overview of the alignment indicates a lack of homology in the N terminal region that extends for further 9 amino acids in the yeast Fbp1p compared to the mammalian homologues 15. This part has been correlated to the glucose-mediated irreversible deactivation of yeast Fbp1p. The Pro1 residue has been implicated in targeting the enzyme to ubiquitin-mediated proteasome degradation in presence of glucose 4,16. The residue Ser11 is linked to cAMP-mediated phosphorylation leading to loss of activity following glucose reintroduction to the medium 17. Structural studies on porcine Fbp1p identified residues 52 to 72, as homologues to residues 63 to 83 in yeast, which form a loop that plays a crucial role in determining the tertiary structure of the tetramer, depending on the presence or absence of AMP and thus mediating AMP allosteric inhibition on the enzyme 18.

Two amino acids of interest in this loop are conserved between yeast and mammalian Fbp1p. The first is Asp68 in mammalian Fbp1p, Asp79 in the yeast homologue, which orients towards the active site, and facilitates the transfer of a proton from the phosphate group 1 of the substrate F1,6P2 to the OH group of the product F6P. The second amino acid is the Asn64, Asn75 in yeast, which in addition to two other amino acids Asp 74 and Glu 98, Asp 85 and Glu 109 in yeast, contributes to the formation of a water-binding site. The water molecule bound to this site indirectly facilitates the nucleophilic attack of an adjacent water molecule on the phosphorus atom of the phosphate group on the position 1 of the substrate which consequently leads to the hydrolysis of the phosphate group and its splitting from the substrate 18.

The stride of residues Asp128 to Ser133 in yeast Fbp1p, or Asp118 to Ser 123 in mammalian Fbp1p, form an evolutionary conserved metal-binding motif. This sequence is largely conserved through pro and eukaryotic evolution; moreover it is encountered in various phosphatases that similarly require divalent cations as allosteric activators. In case of both mammalian and yeast Fbp1p the sequence is comprised of the following residues Asp-Pro-Ile-Asp-Gly-Ser 19. Phe 194 is a conserved residue in both pro and eukaryotes, it lies within a conserved stretch of amino acids. Due to this, in addition to its high bulkiness, we presumed that this residue could be of significance for either the enzyme’s folding or its protein/protein interactions.

The largest region of sequence homology in Fbp1p is on the carboxylic terminus and spans the residues Ala254 to Ser333 of the yeast homologue, and Ala243 to Ser321 of the mammalian enzyme, shows 66% sequence identity between the yeast and mammalian homologues. This region has been linked to the catalytic activity of the enzyme; a notion that has been deployed in recent studies on the human Fbp1p as well 8,20. This c-terminal catalytic sequence contains two evolutionary conserved histidine residues; His265 and His324, both are very likely to comprise a part of the enzyme’s catalytic domain.

Based on the aforementioned data, we screened 9 different mutated versions of yeast Fbp1p each having one of the formerly presented key-residues substituted with an alanine residue through a point mutation, alanine mutagenesis screening, as shown in Table 1.

**Table 1:** Summary of the analyzed mutations and utilized mutagenesis primers.

**Mutations(Altered residue)**	**Mutagenesis primers**	**Abbreviation of the mutated FBP1 form**
Pro1→Ala1	fw: GGATTCTAGAACTAGTATGGCAAC TCTAGTAAA TGGACC rev: GGTCCATTTACTAGAGTTGCCATACTAGTTCTAGAATCC	Pro1
Asn75→Ala75	fw: GTTAGCAGGCGCTTCCGCCTTCACTGGTGACCAGC rev: GCTGGTCACCAGTGAAGGCGGAAGCGCCTGCTAAC	Asn75
Asp79→Ala79	fw: GCTTCCAACTTCACTGGTGCCCAGCAAAAGAAGTTGGAC rev: GTCCAACTTCTTTTGCTGGGCACCAGTGAAGTTGGAAGC	Asp79
Glu109→Ala109	fw: GGTCCTTGTATCTGAAGCACAGGAAGATTTGATCG rev: CGATCAAGTCTTCCTGTGCTTCAGATACAAGGACC	Glu109
Asp131→Ala131	fw: GTGTGTTGTGATCCTATTGCTGGCTCCTCAAATTTGGAC rev: GTCCAAATTTGAGGAGCCACGAATAGGATCACAACACAC	Asp131
Ser133→Ala133	fw: GTGATCCTATTGATGGCGCCTCAAATTTGGACGCCGG rev: CCGGCGTCCAAATTTGAGGCGCCATCAATAGGATCAC	Ser133
Phe194→Ala194	fw: GGTGATGGAGTTGATGGGGCTACCTTAGACACAAACTG rev: CAAGTTTGTGTCTAAGGTAGCCCCATCAACTCCATCACC	Phe194
His65→Ala265	fw: CCATGGTTGCTGATGTTGCCAGGACGTTTCTTTACGG rev: CCGTAAAGAAACGTCCTGGCAACATCAGCAACCATGG	His265
His324→Ala324	fw: GATTTGGTGCCAAGTCATACCGCTGACAAATCTTCTATT rev: AATAGAAGATTTGTCAGCGATATGACTGGCACCAAATC	His325

In the following parts of this manuscript, mutated Fbp1p forms will be referred to using the number and three-letter symbol of the residue that had been replaced with alanine, these abbreviations are listed in Table 1.

### Overexpression levels of Fbp1p mutants

**Figure 2:**
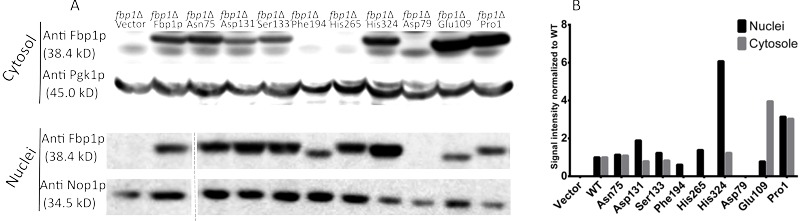
FIGURE 2: Immuno-detection of the over-expressed Fbp1p mutants. **(A)** Western-blot showing the levels of the over-expressed mutants in both cytosolic and nuclear fractions. Over-expression was verified in absence of endogenous Fbp1p expression in *fbp1*∆ strains. PGK1 and NOP1 were utilized as cytosolic and nuclear loading controls, respectively. **(B)** Quantification of the western-blot signals, each band was normalized to the intensity of its loading control, resulting values where then normalized to those of the wt.

Using immuno-blotting, 6 out of the 9 mutated forms of Fbp1p were successfully detected in the cytosolic fraction of the *fbp1*∆ yeast strains in which the mutated Fbp1p forms were over-expressed (Fig. 2A). No cytosolic bands were detected for the Asp79, Phe194 and His 265 mutants. More mutants were successfully detected in nuclear fraction lysates of the different strains with mutated Fbp1p, with only the Asp79 mutant remaining undetected (Fig. 2A). Quantification of band intensities showed that the Asp131, Ser133 and His324 mutants were highly enriched in the nucleus compared to the wild-type (wt) form, and, interestingly, Phe194 and His265 mutants were fully retained in the nucleus (Fig. 2B).

### Catalytic activity of the Fbp1p mutants

In order to screen for the impact of amino-acid substitutions on the catalytic activity of Fbp1p, we over-expressed the mutated cassettes of *fbp1* as well as wt *fbp1*, in BY4741 wt haploid yeast strain. Subsequently we measured the fructose 1,6 bis-phosphatase (FBPase) enzymatic activity in the native protein lysates of the strains over-expressing the mutated forms of Fbp1p and statistically compared them to both cells over-expressing wt Fbp1p and those containing the control vector.

**Figure 3:**
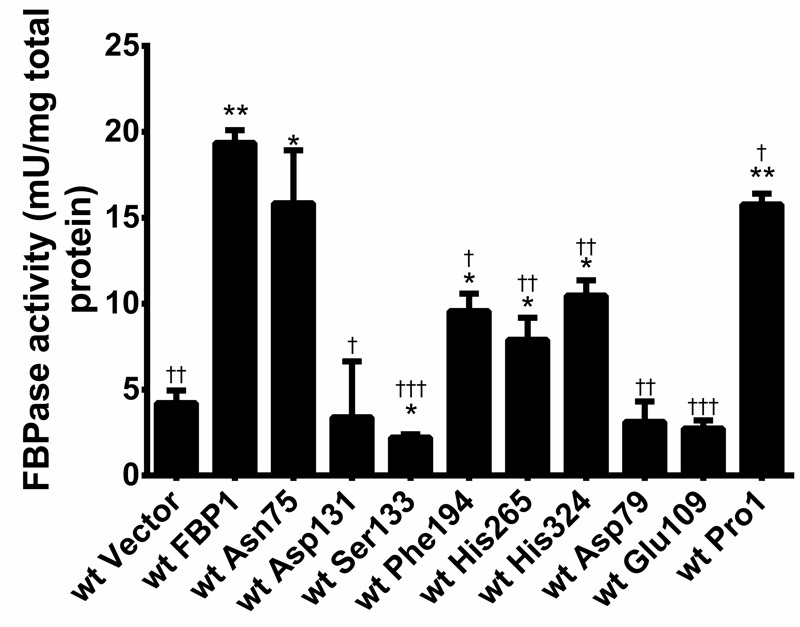
FIGURE 3: Enzymatic activity of the utilized *FBP1* mutants. FBPase catalytic activity was measured in native lysates of wt BY4741 strains with over-expression of wt and mutated *fbp1* cassettes. Measurements were performed in triplets, and error bars represent SD. Statistical significance was determined using student t-test for each of the mutants compared to both cells with control vector and those with over-expression of the wt Fbp1p. (*) Indicates statistically relevant difference compared to empty vector and (†) compared to the wt Fbp1p.

The assay shows that the mutants roughly cluster into 3 groups in respect of their exhibited enzymatic activity. Pro1 and Asn75 showed close-to-wild-type, only slightly reduced activity, nevertheless significantly different; Phe194, His265 and His324 diminished the activity to around 50% compared to the wt; while Asp79, Glu109, Asp131 and Ser133 completely abrogated the activity stemming from over-expression showing a similar activity to extracts of cells transformed with empty vector (Fig. 3).

### Rescue of growth on non-fermentable medium (Drop-test on SDEG)

**Figure 4:**
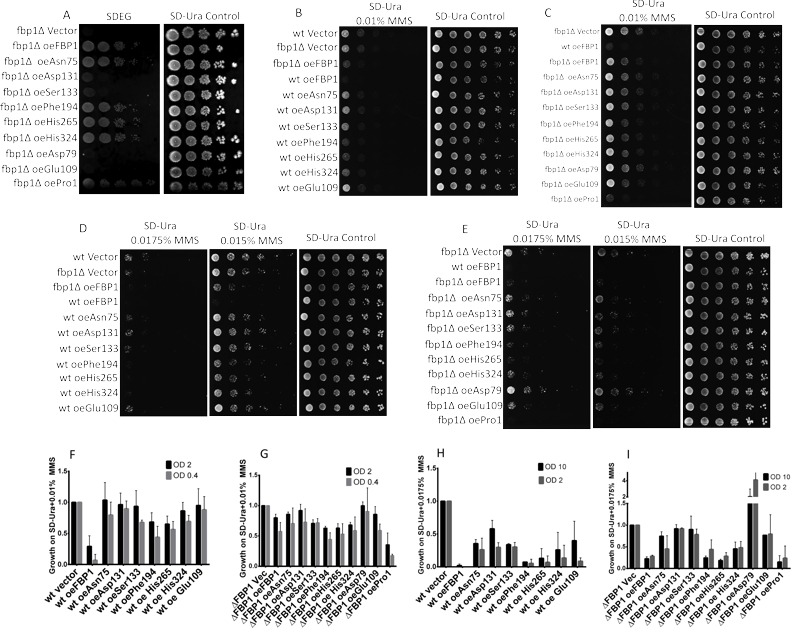
FIGURE 4: Drop-tests on Non-fermentable (SDEG) and MMS-containing agar-media. **(A)** Drop-test on SDEG non-fermentable medium. Wt and mutated *FBP1* cassettes were introduced into BY4741 *fbp1*∆ cells lacking endogenous *FBP1*, the resulting strains were grown in liquid SD-Ura medium, diluted and spotted on SDEG, same suspensions were spotted on SD-Ura agar as a control. Agar-plates were incubated for 48 h at 30°. **(B and C)** Drop tests on SD-Ura agar with 0.01% MMS. Mutants and wt cassettes were introduced into both wt **(B) **and ∆*fbp1* **(C)** BY4741 cells. The resulting strains were grown in SD-Ura and then diluted (1:5) and spotted on freshly prepared SD-Ura containing 0.01%, and on SD-Ura as non-treated control. Agar plates were incubated for 48 h at 30°C. **(D and E)** Drop tests on SD-Ura agar containing 0.015% and 0.0175% MMS. Plates were incubated for 72 h at 30°C. **(F to I)** Quantifications of the spot-intensities of the drop tests on SD-Ura 0.01% MMS second and third spots (OD_600_: 2 and 0.4 respectively) and 0.0175% first and second spots (OD_600_: 10 and 2 respectively). Featured results are taken from three biologically independent replicates, (error-bars: SD; N = 3).

In order to assess the mutants’ potential of rescuing the gluconeogenic pathway in cells lacking *FBP1* expression. The mutant *FBP1* forms were introduced into the *FBP1* knockout strain BY4741-∆*fbp1*. Drop tests on SDEG-medium, containing glycerol and ethanol as exclusive carbon sources, were then performed with the *fbp1*∆ strains over-expressing the Fbp1p mutant (Fig. 4A). Since the enzymatic activity of Fbp1p is indispensable for gluconeogenesis, the determining factor for the growth of each strain on the non-fermentable SDEG medium is the catalytic activity of the Fbp1p mutant expressed.

Four mutants, Asp79, Glu109, Asp131 and Ser133, completely failed to rescue growth on SDEG. The other screened mutants, Pro1, Asn75, Phe194, His265 and His 324, clearly rescued growth on SDEG-agar exhibiting various levels of growth (Fig. 4A).

### Sensitivity to low doses of MMS

Since we had shown previously that exposure to low doses of MMS triggers *fbp1* transcription with the toxic outcome strongly depending on the presence of endogenous Fbp1p, we analyzed the effects of mutations in key residues of Fbp1p on its ability to mediate increased sensitivity to MMS. For this purpose, expression vectors with mutated *fbp1* cassettes were introduced into both wt and Fbp1p-deficient *fbp*1∆ BY4741 cells. Subsequently, the resulting strains were spotted on SD-Ura agar containing MMS concentrations ranging from 0.01% to 0.0175%. These drop tests showed that the catalytically inactive mutants Asp79, Glu109, Asp131, Ser133 and His324 contributed no additional sensitivity towards MMS, and had growth levels comparable to wt cells harbouring control vector (Fig. 4B to I). Three catalytically active mutants Pro1, Phe194 and His265 conferred increased sensitivity toward MMS, when over-expressed in wt or Fbp1p-deficient *fbp1*∆ cells, these mutants impeded growth on MMS compared to the corresponding strains with control vector (Fig. 4B to I). Interestingly, one catalytically active mutant, Asn75, contributed much less additional sensitivity towards MMS compared to wt and to other less catalytically active mutants (Fig. 2B to I). Similar trends were observed in both wt and *fbp1*∆ cells, with all wt-based strains showing less over-all growth with MMS concentrations of 0.01% and 0.0175%. These results consist with our previously reported finding that *fbp1* deletion confers resistance towards low doses of MMS 13. Quantifications of spot intensities of the drop-tests confirmed the eye-observable changes in MMS sensitivity with different Fbp1p mutations (Fig. 4F, G, H and I).

### Over-production of Fbp1p sensitises cells to MMS in liquid medium

**Figure 5:**
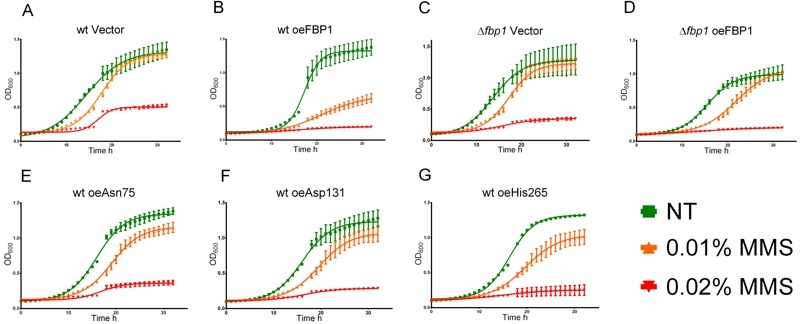
FIGURE 5: Growth curves upon MMS treatment. Figures A to G feature the growth curves of the non-treated and MMS-treated (0.01% and 0.02%) yeast strains in liquid SD-Ura medium. Curves feature the increasing OD_600 _values over-time at 30°C with intermittent shaking, error bars represent SD, N = 3. **(A)** wt Vector,** (B)** wt oe *FBP1*, **(C)** *fbp1*∆ Vector, **(D)** *fbp1*∆ oe Fbp1p, **(E)** wt oeAsn75,** (F)** wt oe Ser133, **(G)** wt oe His265.

To better understand the cytotoxic effects of MMS, we also evaluated its impact on yeast proliferation in liquid media. Cultures were pre-incubated for 3 h and then treated with 0.01% and 0.02% MMS in 96 well plates, subsequently OD_600_ was measured over-time using a plate reader. While treatment with 0.02% MMS resulted in severe inhibition of growth of all tested strains, the impact of 0.01% on proliferation in SD-Ura was clearly influenced by Fbp1p activity (Fig 5A to G). Over-expression of Fbp1p generally reduced growth, however a clear contribution of the endogenous Fbp1p was also observed. Over-expression of Fbp1p in wt cells sensitized them to the lower MMS concentration of 0.01% leading to less over-all growth (Fig. 5A and B). In contrast, the effect of Fbp1p over-expression in cells lacking endogenous Fbp1p was very mild and limited to a temporary inhibition of growth (Fig. 5C and D). Three mutants were tested in comparison to over-expression of wt Fbp1p in the wt cell background. Over-expression of the catalytically active mutants Asn75 and His265 led to a sensitizing effect at 0.01% MMS similar to the effect seen with the wt Fbp1p (Fig. 5E and G), while the inactive mutant Asp131 had no noticeable effect (Fig. 5F).

### The effect of Fbp1p over-production on cell survival depends on its genomic context of *fbp1*

**Figure 6:**
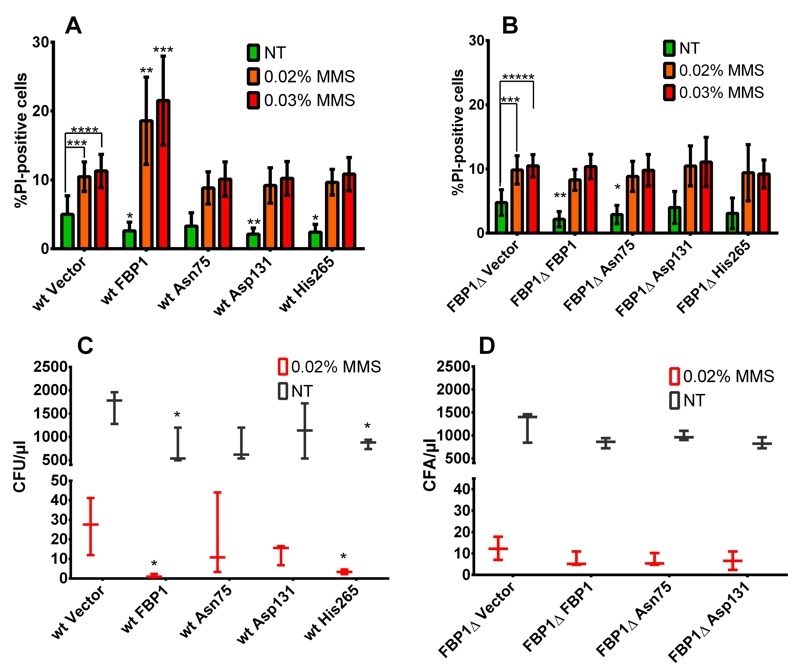
FIGURE 6: Cell survival and colony formation upon MMS treatment. **(A and B)** FACS analysis of PI staining following 12 h of MMS treatment. Bars represent the PI-positive percentage fractions of cells as measured using FACS. Results feature three biologically independent replicates with three technical replicates each. Error bars show the standard deviation. Statistical significance was determined using unpaired student t-test assuming equal variance. (*: p < 0.05; **: p < 0.01; ***: p < 0.001; ****: p < 0.0001). The brackets over the wt Vector and *fbp1*∆ Vector strains the control vector strains show the significance comparing treated and non-treated of the same strain. While significance stars over the mutants represent significance when comparing the results form these strains with those of the control vector. **(C and D) **colony forming on SD-Ura agar following 12 h of MMS treatment in liquid SD-Ura. Results are from 3 independent experiments. Error bars show the standard deviation. Asterisks indicate statistically significant differences compared to the control vector strains. Significance was assessed using student t-test (*: p < 0.05).

We then sought to assess the impact of over-expression of Fbp1p or its mutants on cell survival upon MMS treatment using flow cytometry (FACS) analysis of PI-stained cells. After 12 h of MMS treatment in liquid SD-Ura cultures, all strains had a clear and significant increase in the amount of PI positive cells. Over-expression of wt Fbp1p in wt cells possessing endogenous Fbp1p led to a significant increase in the PI positive fraction upon MMS treatment with both 0.02% and 0.03% concentrations (Fig. 6A), whereas no significant increase in the PI-positive fraction was observed upon over-expression of Fbp1p in cells lacking endogenous Fbp1p (Fig. 6B). We also evaluated the effects of two catalytically active mutants, His265 and Asn75, and the totally inactive mutant Asp131 both in wt and *fbp1*∆ cells. Interestingly, non-of the tested mutants led to any significant difference in the PI-positive fraction after 12 h of MMS treatment (Fig. 6A and B).

Please note, that over-expression of Fbp1p was associated with a significant decrease in the PI-positive fractions of non-treated cells, which was also observed with tested mutants (Fig. 6A and B).

### Over-production of Fbp1p hampers colony-forming capacity upon MMS treatment

To further investigate cell survival upon MMS treatment, we tested the impact of wt and mutated forms of Fbp1p on the colony forming capacity of cells following MMS treatment. Generally, 12 h of 0.02% MMS treatment lead to a two to three orders of magnitude decrease of the colony forming units CFU/µl (Fig. 6C and D). In context of wt cells with endogenous Fbp1p expression, this decrease was strongest when wt Fbp1p or the active mutant His265 were over-expressed (Fig. 6C). In contrast, over-expression of wt Fbp1p in cells lacking the endogenous gene (*fbp1*∆) had no further inhibitory effect on colony forming capacity (Fig. 6D). It is also worth noting that in the wt strain, over-expression of Fbp1p or its active mutant His265 lead to a significant decrease in CFU/µl in the non-treated samples as well, compared to non-treated cells harbouring the control vector.

## DISCUSSION

This work aimed at exploring possible key evolutionary-conserved residues in Fbp1p linked to its apparent multiple-roles in both gluconeogenesis and response to MMS toxicity. In this study, we managed to identify several key residues essential for the enzymatic activity and the role in response to MMS-inflicted damage. The full activity exhibited by the Pro1 mutant completely fits with its reported role as a ubiquitination-mediating residue 16. Replacing Pro1 should abrogate ubiquitination and lead to increased stability, which is clearly reflected in the increased intensity of the western-blot band of this mutant 4. Additionally, when ectopically expressed in *fbp1*∆, the Pro1 mutant showed the highest MMS-sensitisation in the drop tests, even higher than wt Fbp1p, which could be explained by the higher stability of this mutant through evasion of proteasomal degradation. Replacing the residue Glu109 completely abolished the enzymatic activity, along with the ability to grow on non-fermentable carbon sources, however the Asn75 mutant almost maintained full activity compared to the wt, and also rescued growth on SDEG medium. This suggests that Glu109 is significantly participating in binding the catalytically essential water molecule while Asn75 seems to lack an important role in the formation of the water-binding pocket. The complete lack of enzymatic activity when Asp131 or Ser133 are replaced grants validity to their structurally presumed participation in the divalent-cation binding pocket and to the importance of the divalent cation for the activity of this enzyme.

Substituting Phe194 leads to a partial loss in enzymatic activity. This could be due to a direct influence on the catalytic activity. However, this lack of activity could also result from a change in molecular interaction and cellular localization, which was predominantly nuclear for this mutant.

Histidine residues in proximity to active sites on enzymes are usually presumed to contribute to the catalysis due to the amphiphilic nature of their side-chain imidazole ring 32. In our study, altering either His265 or His324 reduced the FBPase catalytic activity by more than one fold; this supports the importance of these residues in facilitating the catalysis at the active site near the c-terminus.

Nevertheless, the activity is not entirely dependent on these residues. In both cases, the enzyme retains sufficient activity, and mediates the ability of growing on SDEG medium in transformed strains.

**Figure 7:**
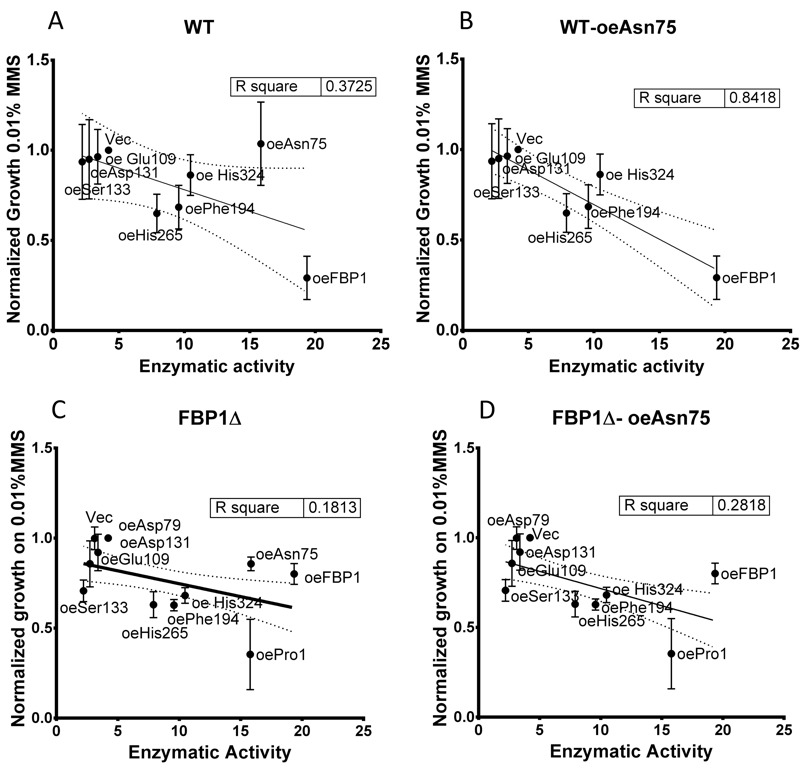
FIGURE 7: Linear correlation between enzymatic activity and growth on SD-Ura agar with 0.01% MMS in drop-tests. **(A)** wt strains; **(B)** wt strains excluding Asn75; **(C)** *fbp1*∆ strains; **(D)** *fbp1*∆ strains excluding Asn75. MMS growth values are taken from the spot-intensity quantification of three independent drop tests on SD-Ura agar with 0.01% MMS. N = 3; error bars represent SD.

For most mutants, the observed sensitivity to MMS correlated to the measured FBPase catalytic activity, and the capacity to grow on SDEG non-fermentable medium. However, two mutants clearly defied this pattern. Asn75 showed full enzymatic activity, and also permitted growth on SDEG medium. However, Asn75 showed much less inhibition of growth on SD-Ura with MMS compared to the wt enzyme and other catalytically active mutants. This effect was verified repeatedly in drop-tests using both wt and *fbp1*∆ strains. Similarly, the His324 mutant, exhibiting almost 50% of the original catalytic activity, did not significantly increase sensitivity towards MMS in the *fbp1*∆ strain. The decoupling of MMS-sensitisation from the FBPase activity in case of the Asn75 is further highlighted by investigating the linear correlation between the measured FBPase activity of the mutants and their quantified growth on 0.01% MMS in drop-tests (Fig. 7). In case of wt cells with intact endogenous Fbp1p, Asn75 lies far from the correlation line out of the confidence interval, showing less negative correlation between its catalytic activity and the growth of its hosting strain on MMS in drop-tests (Fig. 7A). Furthermore, the exclusion of the Asn75 from the analysis, leads to substantial improvement in the linear correlation marked by an increase of R-square value from (0.37 to (0.84 (Fig 7A and B). Thus, the Asn75 and also possibly the His324 decouple the catalytic activity of the enzyme from its MMS-sensitizing role.

Substituting the residue His265 reduced enzymatic activity to less than half of wt Fbp1p, whereas this mutant conveyed even higher sensitivity towards MMS compared to the wt enzyme. In case of this His265 mutant it must be noted that it was enriched in the nuclear fraction and could not be detected in cytosolic extracts by western-blot, which may lead to lower activity in the enzymatic activity assay and may contribute to its increased MMS-sensitizing effect.

Taken together, our observations that for some mutants the catalytic activity of Fbp1p is decoupled from its additional role mediating sensitivity to MMS treatment suggest that these effects are mediated by two at least partially independent sites in the enzyme, one for enzymatic activity and one underlying the MMS-response. This clearly contradicts the alternative model, in which the MMS-sensitizing role of *FBP1* would be directly linked to its catalytic activity and its role in gluconeogenesis.

Hypothesising on more speculative explanations that potentially could clarify the role of *FBP1* in response to MMS treatment, we searched for previously reported interactions of *FBP1* in the literature. A physical interaction with TORC1 was reported in studies analyzing Fbp1p degradation upon glucose reintroduction following long-term glucose starvation 21,22. High throughput screenings also showed a genetic interaction with TOR2 23. TORC1 is a nutrient-sensing protein-kinase complex that responds to nutrient abundance by driving anabolic pathways required for cell growth and proliferation in addition to promoting cell cycle progression 24,25. Under glucose starvation, Fbp1p binds to TORC1, with the latter being essential for glucose-mediated Fbp1p degradation 21,22. These findings suggest a potential scenario in which the Fbp1p expressed in response to MMS treatment also binds to and inhibits TORC1 leading to a more significant inhibition of cell proliferation. Such Fbp1p-mediated loss of TORC1 function would consist with the increased viability upon MMS treatment in absence of Fbp1p, it could also explain the observed uncoupling of the enzymatic activity from MMS-sensitization. Other reported genetic interactions suggest an involvement of *FBP1 *in cell cycle regulation, these include: CDH1 23, a cell-cycle regulated mitotic exit protein, GDH1 26 glycogen phosphorylase postulated to co-ordinate metabolism to cell-cycle, TAF1 27 involved in G1/S progression of cell cycle and ADK1 26, involved in purine metabolism and response to DNA-replication stress. The aforementioned interactions make it very intriguing to functionally probe these suggested interactions and their effects on cell cycle progression in context of DNA damage, an interesting open question we intend to pursue in the near future.

Our findings consistently showed that the outcome of *FBP1 *over-expression is very dependent on its genetic context and the presence of endogenously expressed Fbp1p. This is in line with earlier reports that MMS treatment induces the transcription of *FBP1 *within the first hour of treatment 13. It is also already known that the lack of *fbp1* on a genomic level confers more resistance against MMS treatment 13. In this work we found that the impact of the ectopically expressed Fbp1p on cell survival upon MMS treatment largely depends on the presence of the endogenously expressed enzyme. Whereas over-expression of Fbp1p inhibits growth on SD-Agar MMS in both wt and *fbp1*∆ genotypes, the presence of endogenous Fbp1p seems to be the decisive factor when it comes to the impact of ectopically expressed Fbp1p on cell survival and sustained damage upon MMS treatment, this is made clear by the drastically different outcomes of PI-staining and colony formation of MMS-treated cells with Fbp1p over-expression when using the wt or *fbp1*∆ cells. Additionally our observations support the notion that *FBP1 *delays aging in yeast as evident by the significant decrease in PI-positive fractions of non-treated cells with Fbp1p over-expression compared to the vector control.

**Figure 8:**
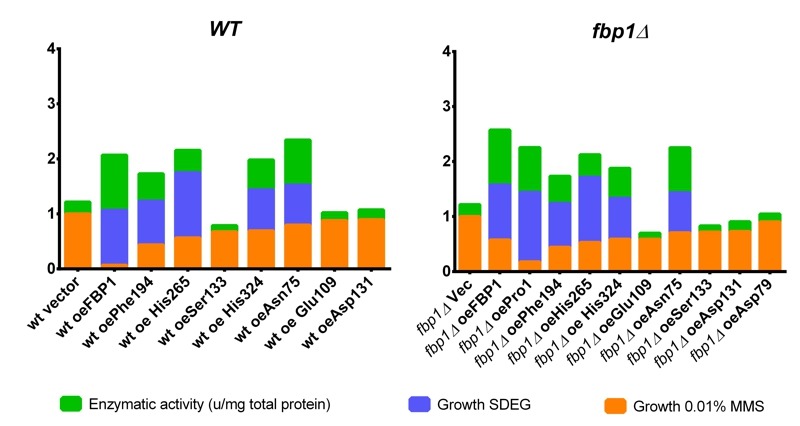
FIGURE 8: Summary figure. This figure summarizes the observed effects of the investigated mutations on FBPase catalysis, growth on non-fermentable SDEG and growth on SD-Ura agar containing 0.01% MMS. Featured are the normalized values corresponding to enzymatic activity measured in the wt-strain background, image quantification of drop-tests on SDEG of the *fbp1*∆-derived strains and averages of three independent replicates of drop-tests on SD-Ura agar with 0.01% MMS with both wt and *fbp1*∆ strains. Enzymatic activity and growth on SDEG were normalized to the strain over-expressing wt Fbp1p, while growth on SD-Ura agar with 0.01% MMS was normalized to the strain harbouring empty vector.

A summarizing figure (Fig. 8) depicts the observed effects of the mutants on three studied phenomena: catalytic FBPase activity, growth on non-fermentable carbon sources (SDEG) and growth on SD-Ura agar with 0.01% MMS in drop tests. The bars in the graph are sorted by decreasing the MMS-sensitivity (increasing values of growth on MMS). The decoupling of MMS-sensitisation from catalytic activity, discussed above, is again clearly visible in this figure. While the Asn75 mutant, catalytically active and conferring growth on SDEG, does not significantly sensitize cells to MMS in both wt and *fbp1*∆ genetic backgrounds, in case of His324, which is also catalytically active and confers growth on SDEG, the decoupling is most clearly visible in the wt strain.

In this work we successfully identified several conserved key residues for the catalytic function and/or the increased sensitivity to MMS and provide a clear separation of both activities. Our observation in yeast fits well with a recent report on FBP1 protein interactions in human tumours, which also seem to be independent of its catalytic activity 8,28.

## MATERIAL AND METHODS

### Utilized yeast strains, media and growth conditions

All yeast strains were cultured and utilized in their haploidic-a form. Strains with the genetic background BY4741 (either wt or ∆*fbp1*) were used to over-express the mutated versions of Fbp1p and screen for the effects of the mutations on enzymatic activity, growth on non-fermentable SDEG medium and sensitivity towards MMS treatment.

Transformed strains were maintained on synthetic minimal medium without uracil (SD-Ura) agar, containing 6.7 g/l yeast nitrogen base, 20 g/l glucose 100 mg/l hestidine, lucien and lysine. Prior to treatments starting cultures were made by inoculating a single colony into 2 ml of SD-Ura liquid medium and incubating at 30°C with shaking overnight.

### Plasmid vectors

Wt and mutated cassettes of FBP1 were over-expressed under the yeast GPD promoter using the high-copy number pRS426 plasmid vector containing *Ura3* as a selection marker in yeast.

### Preparation of *fbp1* mutant plasmids

The plasmid pRS426fbp1containing the wt fbp1 cassette was obtained from previous work performed in our lab 13.

Mutants of *fbp1* were prepared using the PCR-based site-directed mutagenesis method using primers containing the desired point mutations, in combination with a commercially available kit "QuickChange® XL Site-directed mutagenesis kit"(Stratagen). Mutagenesis-PCR reactions were carried out according to the kit’s instructions and 9 mutants of *fbp1* were prepared, each containing a single point mutation leading to the conversion of the codon of the targeted residue into an alanine codon.

The mutated residues, their corresponding mutagenesis primers and symbols used to designate them in this work are featured in table 1.

### Transformation of yeast cells

Competent Yeast cells were prepared and transformed using the Lithium Acetate and heat shock assisted method described by Gietz *et al.* 29.

Afterwards, successfully transformed cells were selected and further cultured on SD-Ura3 medium.

### Protein extraction from yeast cells

Cells from over-night culture in SD-Ura medium were pelleted at 1000 x g. Afterwards pellets were re-suspended in 1 ml 0,8 M NaCl containing 0,2 mg Zymolase and incubated for 30 min at room temperature. The resulting spheroplasts were then spun down and re-suspended in 1 ml 10 mM Tris containing protease inhibitors. Subsequently ca. 100 μl glass beads were added and spheroplasted cells were crushed using the bead-mill homogeniser MM300 (Retsch) at full speed for 10 min followed by 10 min on ice. This homogenization/cooling cycle was repeated three times and lysates were obtained.

Nuclei were isolated form the rest of the lysates using differential centrifugation at 1000 x g for 5 min. The supernatant containing the cytosolic fraction of the lysate was isolated and stored at -20°C, and the yielded pellet with the nuclear fraction was resuspended in 0,5 ml 10 mM Tris then centrifuged at 300 x g to remove the cell debris. The remaining supernatant after this step contains the floating nuclei and was isolated and stored at -20°C. Both nuclear and cytosolic fractions were analyzed using SDS-Page followed by western blotting to investigate the presence of the various mutated forms of Fbp1p in transformed strains containing their respective over-expression vectors.

### Immuno-blotting for detection of Fbp1p and its mutant forms

Denatured cytosolic and nuclear protein extracts were resolved using SDS-Page with 10% poly-acrylamide gels. Separated proteins were subsequently transferred onto PVDF membranes by semi-dry blot. Following blocking in 5% BSA at 4°C over-night, membranes were incubated with the primary polyclonal anti-FBP1 antibody in 5% BSA solution for 1 hour at room temperature. Subsequently membranes were washed 3 times with TBS-tween and then incubated with a 1:10000 diluted solution of anti-rabbit secondary antibody. After 5 washing steps membranes were developed using the Chemi-luminescence reagent Western-Lighting, which consists of the reagents oxidizing and enhanced luminol mixed in 1:1 ratio. After 1 min of developing the membranes the signal was visualized using Fuji LAS-3000 imaging system and the AIDA image analyzing software.

The utilized anti yeast-FBP1 antibody is directed against the internal region of the protein, and was granted to our lab as courtesy of the group of Prof. Dr. Dieter Wolf at University of Stuttgart.

### Enzymatic assay of FBPase catalytic activity

Total fructose 1,6 bis-phosphatase activity was measured using cytosolic protein extracts of over-night cultures of yeast cells as described by Skalecki *et al.* (1995) 30. The general principle of the assay depends on coupling FBPase catalysis to the formation of NADPH through the phospho-glucose isomerase PGI and glucose-6-phosphate dehydrogenase G6PDH present in the reaction mix. Dephosphorylation of fructose 1,6 bis-phosphate by Fbp1p yields Fructose 6 phosphate, which is then converted into glucose 6 phsophate by PGI. G6P is then oxidized by G6PDH with NADP+ getting reduced to NADPH. Thus the ratio of the yielded F6P molecules and the NADPH is 1:1. NADPH has an absorption peak at 340 nm, this absorption is then kinetically measured every 2 min for 20 cycles and then the total FBPase catalytic activity is estimated by the increase in absorption at 340 nm over time, using the following equation:

**Figure:**
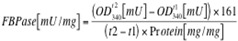


### Growth measurements in liquid cultures

Over-night cultures were first diluted to an OD_600_ of 0.2, and shaken for 3 h. Subsequently cultures were transferred into 96 round-bottom well-plates, 200 µl per well. Of each used cell type, three wells were treated with 0.02% MMS, and three wells with 0.01% MMS while four wells were kept untreated. The 96 well plates were then incubated at 30°C inside the microplate reader (Tecan-Ultra). OD_600 _of each well was measured every hour for 32 h, 15 min of shaking within the plate reader preceded each measurement.

### Drop-tests

Growth on non-fermentable carbon sources, drop test on SDEG medium

Starting from Mid-Log phase cultures, cell suspensions with the following dilution factors were prepared via serial dilution (1:10).

1:1; 1:10; 1:100; 1:1000; 1:10000; 1:100000.

Subsequently, 4 µl of each cell suspension of the examined yeast strains were adjacently dropped onto SDEG-agar. Agar plates were incubated for 48 h at 30°C and then imaged to assess the growth of the strains over-expressing either wt or mutant versions of Fbp1p.

#### Sensitivity to MMS treatment

Starting from over-night cultures, cell-suspensions OD was adjusted to 10, and these suspensions were serially diluted (1:5) to yield suspensions with the following OD600 (10; 2; 0.4; 0.08; 0.0016; 0.00032). 4 µl of each suspension was then spotted onto freshly prepared SD-Ura plates containing varying concentrations of MMS (0.01% to 0.0175%).

The plates with 0.01% MMS, and those with 0.015% or 0.0175% MMS were incubated at 30°C for 48h and 72h, respectively.

#### Drop-test Image-Quantification

Spot-intensities from drop-tests were quantified using ImageJ, spot intensities from MMS plates were then normalized to those of the non-treated control, and values from mutants were normalized against the strain with wt Fbp1p.

### Cell survival upon MMS treatment

#### Quantification of unviable cell fractions and colony formation assay upon MMS treatment

Unviable cells were detected using Propidium Iodide (PI). PI is a red-fluorescent dye that stably binds the DNA once it enters cells. While healthy cells retain PI out of their intact membranes, disrupted membranes of severely damaged and/or dead cells are permeable to PI. Therefore PI can be used to differentiate the unviable and severely damaged cells from the rest of the healthy cell population 31.

Over-night cultures in SD-Ura were first diluted to an OD_600_ of 0.2 and incubated for 5 h till it reached mid-log phase. Each culture was then split into three parts, non-treated and treated with 0.02% and 0.03% MMS. Following 12 h of shaking at 30°C, cultures were diluted and cell density of all samples was adjusted to OD of 0.5. Subsequently the adjusted suspensions were used for both PI-staining and colony formation assay. For PI-staining cell suspensions were pipetted in triplicates in a round-bottom 96 well-plate and then simultaneously stained with propedium-iodide at a concentration of 125 µg/ml and shaken for 10 min in the dark. Afterwards, cells were analyzed using the automatic FACS-guava system. PI-positive sub-populations were determined and calculated as a percentage of the whole cell population. The experiment was repeated 3 times, each with three technical replicates.

For the colony formation assay, the adjusted cell-suspensions of non-treated samples were diluted 1:1000 (three step serial dilution 1:10) then 50 µl of each diluted sample were spread on an SD-Ura agar plates. Adjusted suspensions of cells treated with 0.02% were diluted 1:10 and then 50 µl of each diluted sample was spread on an SD-Ura agar plate.

Plates were incubated for 48 h at 30° then colonies were manually scored.

This experiment was repeated three times independently.

Ali Ghanem is supported by a doctoral fellowship from the German Academic Exchange Service DAAD
